# Accelerated Simple Preparation of Curcumin-Loaded Silk Fibroin/Hyaluronic Acid Hydrogels for Biomedical Applications

**DOI:** 10.3390/polym15030504

**Published:** 2023-01-18

**Authors:** Mohamed Chaala, Fatima Zohra Sebba, Marta G. Fuster, Imane Moulefera, Mercedes G. Montalbán, Guzmán Carissimi, Gloria Víllora

**Affiliations:** 1 Laboratoire de Chimie Physique Macromoléculaire, Département de Chimie, Université Oran1 Ahmed Ben Bella, B.P 1524, El-Menaouer, Oran 31000, Algeria; 2 Chemical Engineering Department, Faculty of Chemistry, Regional Campus of International Excellence “Campus Mare Nostrum”, University of Murcia, 30071 Murcia, Spain

**Keywords:** silk fibroin, hyaluronic acid, hydrogel, curcumin, cellular proliferation

## Abstract

The development of new biomaterials from natural fibres in the field of biomedicine have attracted great interest in recent years. One of the most studied fibres has been silk fibroin produced by the Bombyx mori worm, due to its excellent mechanical properties and its biodegradability and bioavailability. Among the different biomaterials that can be prepared from silk fibroin, hydrogels have attracted considerable attention due to their potential use in different fields, such as scaffolding, cell therapy and biomedical application. Hydrogels are essentially a three-dimensional network of flexible polymer chains that absorb considerable amounts of water and can be loaded with drugs and/or cells inside to be used in a wide variety of applications. Here we present a simple sonication process for the preparation of curcumin-hyaluronic acid-silk fibroin hydrogels. Different grades of hydrogels were prepared by controlling the relative amounts of their components. The hydrogels were physically and morphologically characterised by Fourier transform infrared spectroscopy (FTIR), X-ray diffraction (XRD), thermogravimetric analysis (TGA) and field emission scanning electron microscopy (FESEM) and their biological activity was tested in terms of cell viability in a fibroblast cell line.

## 1. Introduction

In recent years, the development of biocompatible hydrogels for applications in the biomedical field has gained much attention [1]. Their three-dimensional structure which provides support, and their ability to absorb large amounts of water while maintaining their structural integrity are useful features for these applications. In addition, their high porosity allows the for rapid diffusion of small molecules. Recently, the use of hydrogels for tissue engineering and wound healing applications has driven important research efforts [2,3,4]. In vivo, the cells grow, divide, perform their functions, communicate with others, and migrate; these functions are supported by the extracellular matrix (ECM), which provides mechanical support as well as physicochemical signals to the cells to perform these functions. The ECM is composed of fibrous proteins (mainly laminin, collagen, and fibronectin) whose chains form physical networks that provide mechanical support, and proteoglycans that occupy the interstitial sites of this polymeric network [5]. Thus, hydrogels have been widely accepted as near prototypes of the ECM and have been found to be suitable three-dimensional matrices for cell growth that provide a suitable biochemical environment in which cell-matrix interactions can occur [6]. To achieve these requirements, multi-component hybrid hydrogels are a more promising option because, by combining of the appropriate materials, it easier to the properties of the hydrogel [7,8].

A key concept in tissue engineering is the selection of the appropriate material to design and produce an adequate hydrogel that does not induce any or minimal immune reaction from the recipient. In this context, biomaterials have the ability to coexist and interact in the presence of specific tissues or physiological systems such as blood, interstitial fluids, and immune cells and molecules without inflicting intolerable damage [4]. Silk fibroin (SF) Bombyx mori is a fibrous protein and a natural biopolymer extracted from silk cocoons. It has been widely used for designing of biomaterials due to its relatively inexpensive nature, wide availability and excellent properties, such as biocompatibility, environmental stability, non-toxicity and a controllable rate of biodegradation [9,10]. Compared to other naturally occurring fibres, SF occupies a special position in the preparation of hydrogels due to its peculiar properties. In addition to being a good support for cell growth and proliferation, SF-based biomaterials do not trigger activation of immune response and have a slower degradation, which allows them to support neo-forming tissues for a long duration. Furthermore, the degradation products of SF-based materials have been shown to be harmless to the human body. In addition, not only can the properties of SF be tailored by chemical and physical modifications, but they can also be modulated as need to the requirements by genetic engineering [5]. Recently, SF-based hydrogels have received great interest in the application of tissue engineering, wound repairing and drug delivery therapies [5,11,12,13,14,15].

Hyaluronic acid (HA) is a natural non-immunogenic polysaccharide that is a unique component of the ECM where it plays a vital role in cell-ECM interactions [16]. Naturally, it is a polyanion composed of repeating disaccharide units of β-1,4-d-glucuronic acid–β-1,3-N-acetyl-d-glucosamine [17]. This polymer has been explored for various biomedical applications due to its good biocompatibility and promotion of soft tissue regeneration via its water retention and bioactivity sites that allows good penetration and diffusion of small molecules into HA hydrogels [1]. However, HA biomaterials have poor biomechanical performance, rapid degradation and poor cell adhesion that limit their applications [18]. For this reason, combining HA with protein materials such as SF to mimic the composition and structure of ECM is an excellent option for synthesising SF/HA hydrogels [1,16,17,18,19,20,21,22,23,24]. In this way, a significant improvement in the mechanical properties, biocompatibility and bioactivity of SF/HA biomaterials is achieved, increasing their capability for tissue regeneration [19].

SF/HA hydrogels have been found to be suitable for the storage and controlled release of drugs. Elia et al. [21] loaded SF/HA hydrogels with common anti-inflammatory drugs such as dexamethasone, hydrocortisone, 6α-methylprednisolone, cortisone, prednisolone, and prednisone, and found that the drugs were released in a sustained manner. Wang et al. [22,23] synthesised a composite double-network hydrogel of SF and tyramine-modified HA and used two model drugs (trypan blue and methylene blue as anionic and cationic drugs, respectively) for drug delivery assays. They found that the drug loading and release behaviours of the composite hydrogel can be well controlled by changing the pH value and salt concentration of the soaking solutions. Ziadlou et al. [24] studied the release of small hydrophobic anti-inflammatory and anabolic drugs (vanillic acid and epimedin C) from SF/HA hydrogels, revealing their potential application in cartilage regeneration. Yan et al. [1] loaded SF/HA hydrogels with rhodamine B as a model drug and found a release profile with the features of early release concentration and sustained slow release, which was suitable for the application of drug delivery. However, studies on this matter are still scarce. More specific studies could contribute to the development of systems in which therapeutically relevant drugs can be released in specific tissues or organs. In in tissue engineering and wound healing, the presence of specific drugs in hydrogels may be a successful strategy.

Curcumin (1,7-bis(4-hydroxy-3-methoxyphenyl)-1,6-heptadiene-3,5-dione) is a natural yellow-orange compound that is the main phenolic pigment extracted from turmeric, the powdered rhizome of Curcuma longa [25]. Curcumin is known for its anti-inflammatory, anti-cancer, anti-oxidant, anti-bacterial, anti-viral, and anti-fungal activities [26,27,28]. These characteristics make curcumin a good candidate for use in biomedical and particularly in wound healing applications [29,30,31]. In addition, curcumin also exhibits photodynamic properties that have been exploited to improve its antimicrobial efficacy [32]. This is because curcumin enhances epithelial regeneration, fibroblast proliferation, vascular density, collagen deposition and reorganisation [33]. However, curcumin has some drawbacks in its biomedical application, such as low bioavailability, low aqueous solubility, rapid degradation in physiological fluid and formation of aggregates in intravenous solution [25,26,27,28,29,30,31,32,33,34]. To address these issues, the development of novel curcumin delivery strategies is required and the incorporation of curcumin into a hydrogel matrix can be used in order to achieve longer circulation time, better permeability and stability and thus higher pharmacological activity [34]. Few authors have studied the suitability of curcumin-loaded SF hydrogels [34,35,36] and curcumin-loaded HA hydrogels [37,38] for biomedical purposes with promising results, but, to date, the application of dual curcumin-loaded SF/HA hydrogels has not been explored. Curcumin has been used for its therapeutic properties and there is no evidence that it has an additional role, although in the case of other hydrogels or aerogels composed of other biopolymers, such as collagen or chitosan, it has been described that curcumin may affect the gelation process and confers a three-dimensional microstructure that improves cell adhesion and proliferation [39,40]. As mentioned before, an improvement of the physical and biological properties of the hydrogel is expected when both biopolymers, SF and HA, are used together.

In the present study, SF/HA, curcumin-loaded SF, and curcumin-loaded SF/HA hydrogels are prepared by a simple sonication method. They were physically and morphologically characterised by Fourier transform infrared spectroscopy (FTIR), X-ray diffraction (XRD), thermogravimetric analysis (TGA), and field emission scanning electron microscopy (FESEM). In addition, its biological activity was tested in terms of cell viability in a fibroblast cell line (L929). The main objective of this work is to determine the potential therapeutic effect of hydrogels for biomedical applications.

## 2. Materials and Methods

### 2.1. Materials

Bombyx mori silk cocoons were reared in the sericulture facilities of IMIDA (Murcia, Spain) and raised on a diet of natural fresh leaves of *Morus alba* L. To extract the SF, the raw silk cocoons were shredded in a mill to a particle size of 1 mm, and then boiled in a 0.1 M Na_2_CO_3_ aqueous solution for 120 min to remove sericin, waxes, and impurities. The remaining water-insoluble SF was rinsed thoroughly with ultrapure water and air-dried in a fume hood until constant weight (approximately 24 h). Hyaluronic acid sodium was purchased from Monteloeder Digital Nutracetics (MW 8 × 10^5^ Da, >90% purity). Curcumin (99% purity) was purchased from ChromaDex (Irvine, CA, USA). Purified water (18.2 MΩ·cm at 25 °C; from a Millipore Direct-Q1 ultrapure water system, Billerica, MA, USA) was used throughout. All other chemicals and solvents were of analytical grade and were used without further purification.

### 2.2. SF Dissolution

SF was dissolved in 9.3 M LiBr according to the following protocol. First, the 9.3 M LiBr solution was heated to 60 °C, and then SF was added to obtain a 20 wt.% SF solution. The sample was stirred at 60 °C for 6 h to obtain a yellowish solution. The solution was then placed in a dialysis bag (12 kDa) for 72 h with distilled water to remove the lithium salt. The water was changed every 3 h until a conductivity of less than 2 µS/cm (like that of distilled water) was reached. Finally, the solution was centrifuged to eliminate SF precipitates and concentrated up to a concentration of 5 mg/mL and 10 mg/mL. Both solutions were stored at 4 °C.

### 2.3. Preparation of SF/HA Hydrogels

HA was dissolved in 1 mL of the 5 mg/mL SF solution with the final SF/HA mass ratios of 100/0, 80/20, 60/40, 50/50 and 40/60. The SF/HA solutions were then sonicated for 20 s with an amplitude of 30% in an Eppendorf tube to induce the sol-gel transition and the hydrogels were obtained. They were then lyophilised.

### 2.4. Preparation of Curcumin-Loaded SF Hydrogels

25 mg of curcumin was dissolved in 5 mL of NaOH (0.1 N) and several dilutions were made to obtain a curcumin solution with concentrations of 0.66, 1.25 and 2.5 mg/mL. Then, a fixed volume of 0.5 mL of the 10 mg/mL SF solution was mixed with a fixed volume of 0.5 mL of the diluted curcumin solutions to obtain hydrogels with final SF/curcumin mass ratios of 16/1, 8/1, 4/1 and 2/1. The SF/curcumin solutions were sonicated for 20 s with an amplitude of 30% in an Eppendorf tube to induce the sol-gel transition and the hydrogels were obtained. They were then lyophilised. A sample blank without curcumin was also prepared with this protocol.

### 2.5. Preparation of Curcumin-Loaded SF/HA Hydrogels

Curcumin-loaded SF/HA hydrogels were prepared following a procedure like that described in 2.4. First, curcumin solutions in NaOH (1 N) of concentrations of 1.25 and 2.5 and 5 mg/mL were prepared. Then, a fixed volume of 0.5 mL of the 10 mg/mL SF/HA (80/20, 50/50 and 40/60) solutions were mixed with a fixed volume of 0.5 mL of the curcumin solutions to obtain hydrogels with final (SF/HA) curcumin mass ratios of 8/1, 4/1 and 2/1. The (SF/HA)/curcumin solutions were sonicated for 20 s with an amplitude of 30% in an Eppendorf tube to induce the sol-gel transition and the hydrogels were obtained. They were then lyophilised. The Table 1 present the composition and nomenclature of the samples prepared in this work.

### 2.6. Swelling Properties Test

A dried gels were submerged in deionized water at 25 °C for 24 h. After excess water was removed from the gel surfaces, the wet weight of the gels was determined. The swelling ratio and the water uptake in the gels were calculated by Equations (1) and (2) respectively as follows:

Swelling ratio (g/g) = (W_s_ − W_d_)/W_d_, (1)

Water uptake (%) = [(W_s_ − W_d_)/W_s_] × 100 (2)

where W_s_ and W_d_ are the weights of swollen and dried gels, respectively.

### 2.7. Study of the Kinetic Release of Curcumin from the Different Hydrogels

**Figure 1:**
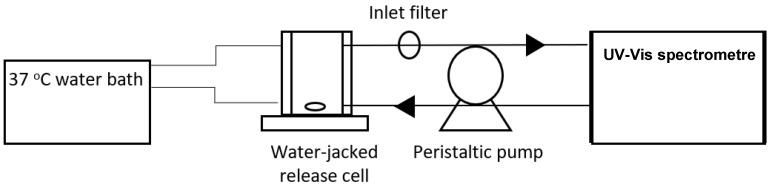
Schematic of the experimental set-up release apparatus.

The in vitro kinetic release of curcumin from the different hydrogels was tested in a self-made USP type 4 flow-through cell apparatus. The device is schematised in Figure 1. The hydrogel was introduced with 50 mL of PBS (pH 7 with 0.5% m/V of Tween 80) in a water-jacked vessel set at 37 °C. The mass of the introduced hydrogel was varied to keep the mass of curcumin constant. For instance, 18, 12 and 22 mg of hydrogels B3, C4 and C9 were weighed, respectively, to maintain 2 mg of curcumin. A peristaltic pump was used to recirculate the release media from the release vessel, through a 0.45 µM nylon filter and the measuring cell at 15 mL/min. The absorbance at 421 nm was measured every 30 s until the end of the experiment with a UV-Vis spectrometer (Reigol, CHINATOWN).

### 2.8. Attenuated Total Reflectance Fourier Transform Infrared Spectroscopy (ATR-FTIR)

The infrared absorption spectra of the hydrogels were recorded on an iS5-Nicolet Fourier-transform infrared spectrometer (Thermo Fischer Scientific, Waltham, MA, USA) equipped with a Deuterated Triglycine Sulfate detector and a 1-reflection, 45° angle of incidence diamond ATR accessory (iD7 ATR module, Thermo Fischer Scientific, Waltham, MA, USA). Each measured spectrum was the average of 64 scans at a data collection rate of 0.47 scans per second. OMNIC Software v9.9.471 (Thermo Fischer Scientific, Waltham, MA, USA) was used to control and process the spectral data. Interferograms were recorded at a resolution of 2 cm^−1^ with a zero-filling factor of 2 in the range of 4000–400 cm^−1^, and Fourier-transformed using the Blackman-Harris 3-term apodization function. A background spectrum without a sample with the same number of scans was collected before each measurement.

### 2.9. Field Emission Scanning Electron Microscopy (FESEM)

To observe the morphology of the hydrogels, an FEI SciosTMmicroscope (Thermo Scientific, Waltham, MA, USA) was used. Cross sections of the lyophilised hydrogel samples were deposited on a mica plate and coated with a thin layer of gold. The mica discs were pre-treated by removing the top layers with scotch tape three times before placing the sample.

### 2.10. X-ray Diffraction (XRD)

X-ray powder diffraction (XRPD) was performed with a D8 Advance diffractometer in Bragg-Brentano geometry (Bruker, Karlsruhe, Germany) with CuKα radiation, 40 kV, 30 mA, and a 1-dimensional LynxEye detector with a 2° window. The primary optics consisted of a 2° Soller slit, a 1 mm incidence slit and an anti-scattering screen that reduces radiation scattering at low angles. The secondary optics included a 3 mm anti-scattering slit, a nickel filter and a 2.5° Soller slit.

For the X-ray analysis, samples were disaggregated in an agate mortar and placed in a 0.5 mm Si sample holder. The samples were passed through a range of 5 to 45° at 2θ, 0.05° intervals, 2 s/stage and 30 rpm rotation.

### 2.11. Thermogravimetric Analysis (TGA-DTA)

The thermal properties of SF were measured using a thermal gravimetric analyser (TA instruments, SDT 2960 simultaneous DSC-TGA, Waters LLC, Champaign, IL, USA) in the temperature range of 25–800 °C at a heating rate of 10 °C/min under an inert nitrogen atmosphere in an open bin. The weight loss was recorded and plotted against temperature for thermogravimetric analysis (TGA) and differential thermal analysis (DTA).

### 2.12. In Vitro Cell Response

#### 2.12.1. Cell Culture

A mouse lung fibroblast cell line (L929) from American Type Culture Collection (ATCC, Manassas, VA, USA) was selected to evaluate the cytotoxicity and biological activity of the hydrogels. L929 was sub-cultured in Dulbecco’s Modified Eagle Medium (DMEM) with a low content of glucose (1 g/L) supplemented with fetal bovine serum (FBS) in a proportion of 0.1 g/mL, 1 mM glutamax, 1 mM pyruvate and 1% antibiotics (penicillin-streptomycin). Cells were incubated at 37 °C in a 5% CO_2_ atmosphere and trypsinised prior to passage using a solution of 0.25% trypsin-0.25 mM ethylenediaminetetraacetic acid (EDTA). The medium was changed twice a week. The cells were checked for absence of mycoplasma before and after the experiments.

#### 2.12.2. Cell Viability

The cytotoxicity of the SF/HA, curcumin-loaded SF and curcumin-loaded SF/HA hydrogels was detected in L929 by MTT assay. In addition, some considerations were taken into account to be able to detect significant changes in absorbance at 560 nm without interference, such as the use of 48-well plates to increase the number of cells in the assay and the use of media without phenol red. Every two days, the medium was changed to fresh medium.

Cell viability of hydrogels was assessed at days 1, 3 and 7. Dried hydrogels were sterilised by ultraviolet irradiation for 30 min. Small pieces of hydrogels were placed in 48-well plates with 250 µL of FBS for 48 h at 37 °C, 5% CO_2_. After FBS was removed, 3 × 10^4^ cells/well were seeded (500 µL final volume) and allowed to incubate according to the time of exposure to the hydrogel.

After treatment, the media was removed and 500 µL of MTT (3-(4,5-dimethylthiazol-2-yl)-2,5-diphenyltetrazolium bromide) solution at a final concentration of 1 mg/mL was added and left in in the dark for 4 h, after which MTT was removed and 250 µL of dimethyl sulfoxide (DMSO) was added. Absorbance was measured on a microplate reader (FLUOstar Omega) spectrophotometer at 560 nm. Each sample was tested in three independent sets with triplicate points. Three controls (hydrogel only, cells only and culture medium only) were added to each plate.

#### 2.12.3. Cell Morphology

The dried hydrogels were cut into small slices and sterilised at 312 nm in 48-well plates. They were then hydrated with 250 μL of FBS for 48 h. The FBS was removed and a suspension of 30,000 L929 cells per well was added. After 72 h, cells were observed by using a Leica inverted microscope: mod DMI1 with image acquisition system.

### 2.13. Statistics

Data were presented as mean ± SD (standard deviation), calculated from three independent samples per condition by using GraphPad Prism 8.0.1 software (GraphPad Software, San Diego, CA, USA). Since normality (Kolmogorov-Smirnov, *p* > 0.05) and homoscedasticity (Levene, *p* > 0.05) were met, statistical significance was determined using Tukey’s parametric test (*p* < 0.05) and ANOVA (*p* < 0.05) for comparisons of two or more groups, respectively.

## 3. Results and Discussion

### 3.1. Characteristics of Hydrogels

**Figure 2:**
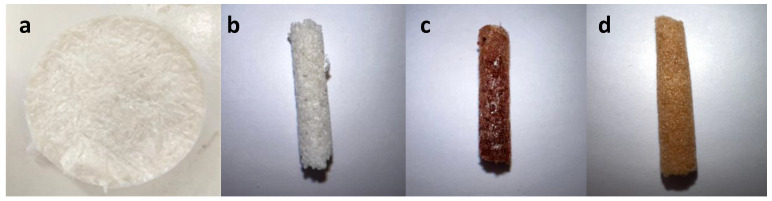
Macroscopic appearance of (**a**) sample SF, (**b**) sample A2, (**c**) sample B3 and (**d**) sample C4.

The macroscopic appearance of hydrogel samples SF (a), A2 (b), B3 (c) and C4 (d) are shown in Figure 2.

From the point of view of the macroscopic appearance of the obtained hydrogels, the most remarkable is the color of the hydrogels containing curcumin as can be seen in Figure 2. Other authors [19] suggest that the addition of HA significantly improves the hardness and other mechanical properties of composite hydrogels through the superhydrophilicity and supramolecular structure of HA macromolecules.

### 3.2. Silk Fibroin Gelation Time

**Figure 3:**
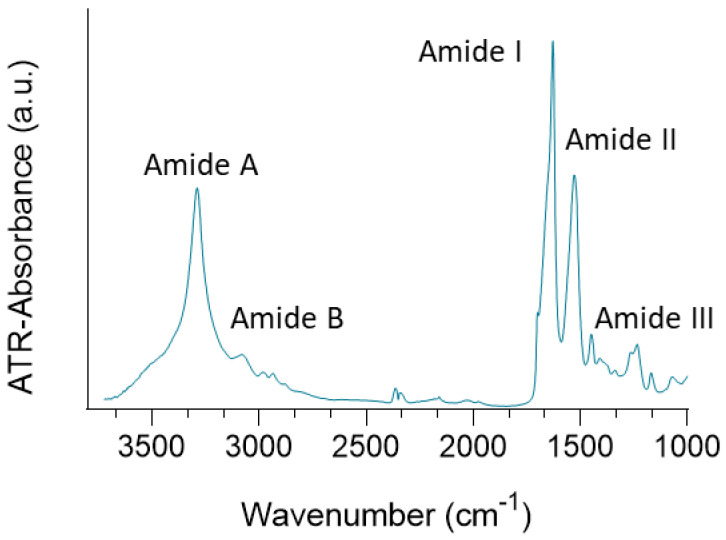
ATR−FTIR spectra of SF fibre.

The SF fibres obtained by the degumming method were analysed by infrared spectroscopy. Figure 3 shows the spectrum obtained, in which the typical bands of SF fibres are observed. Around 3310 and 3270 cm^−1^ is the amide A band, produced by the stretching of the NH group. Around 3100 and 3030 cm^−1^ is the amide B band, produced by the vibration of the CH group. The amide I band, produced mainly by the vibration of the stretching of the C=O group, is observed at approximately 1650 cm^−1^, with its maximum at 1627 cm^−1^. Amide mode II is the out-of-phase combination of the in-plane strain of the NH group and the CN stretching vibration, with minor contributions from the in-plane strain of the CO group and the CC and NC stretching vibrations. For the SF fibre, this band is observed at 1528 cm^−1^. Finally, at 1251 cm^−1^ the amide III band is observed, which arises from the combination of NH group bending and CN group stretching with small contributions from the CO group [41].

**Figure 4:**
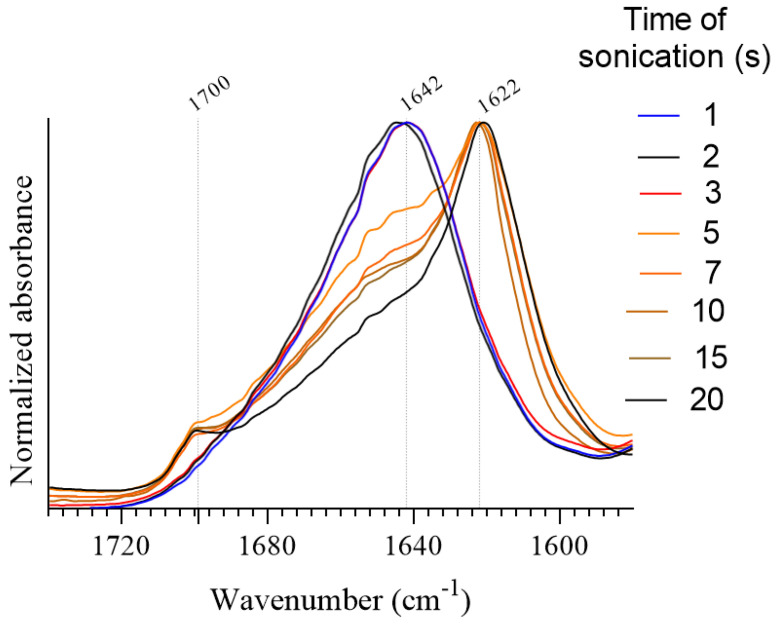
Amide I and II bands of silk fibroin.

The ultrasound time at which the SF solution changed from solution to a non-mobile state (gel) was determined by the evolution of the random coil structure to β-sheets by measuring the ATR-FTIR spectrum. SF samples of 3 mg/mL were sonicated for 1, 2, 3, 5, 7, 10, 15 and 20 s. As shown in Figure 4, with increasing sonication time, there is a gradual shift of the absorption maximum from approximately 1642 cm^−1^ to 1622 cm^−1^. This evolution occurs due to the transition from irregular and type-II β-turns to β-sheets [42]. The samples can be classified into 3 groups based on the position and shape of their amides I bands, which can be related to the sonication time. Samples sonicated for 3 s or less show a broad amide I band with its maximum at 1638–1642 cm^−1^. The position of the absorbance maximum is assigned to irregular and β-sheet structures [43]. When the samples are sonicated for 20 s or more, a decrease in absorbance at 1642 cm^−1^ is observed, while a sharp increase in absorption is seen at 1622 cm^−1^ along with the appearance of a weak band at 1700 cm^−1^. The appearing bands correspond to the main absorption component of antiparallel β-sheets and the high-wavenumber component, respectively. Finally, the amide I band of the samples sonicated between 3 and 20 s shows a mixture of the latter described groups, which is a sharp maximum at 1622 cm^−1^ corresponding to β-sheets with a broad shoulder at 1642 cm^−1^ assigned to irregular and type-II β-turns structures. This indicates that the transition from irregular and type-II β-turns structures to β-sheets is induced by the energy provided by sonications. In the experimental conditions, at least 5 s of sonication were needed to induce a noticeable increase of β-sheets to support the gelation of the solution.

### 3.3. Structure of the Composite Hydrogels

**Figure 5:**
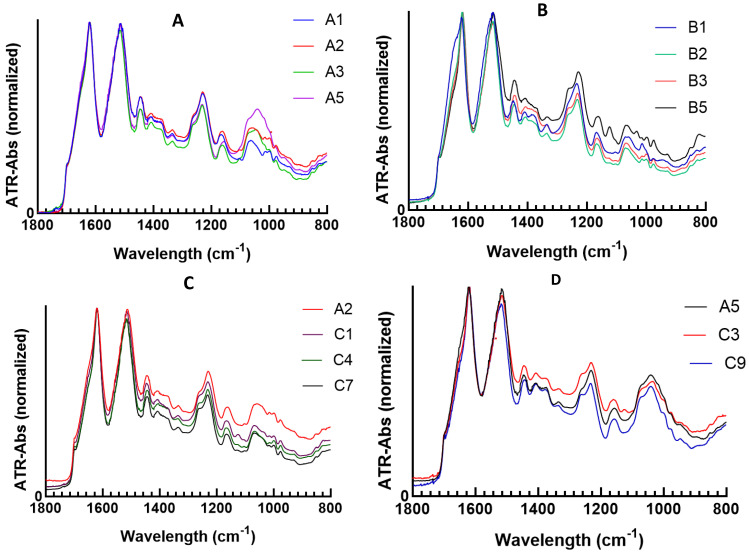
ATR−FTIR absorbance spectra of (**A**) SF/HA hydrogels (A1, A2, A3, A5), (**B**) SF/curcumin hydrogels (B1, B2, B3, B5); (**C**) SF/HA/curcumin hydrogels with SF/HA = 80/20 and SF/curcumin 0, 2/1, 4/1 and 8/1 (A2, C1, C4, C7) and (**D**) SF/HA/curcumin hydrogels with SF/HA = 40/60 and SF/curcumin 0, 2/1, and 8/1 (A5, C3, C9).

To characterise the chemical structural changes in the preparation process of the composite hydrogels, the samples were analysed by ATR-FTIR spectroscopy after sonication. As shown in Figure 5, peaks appear around 1625 cm^−1^, 1515 cm^−1^ and 1230 cm^−1^ which were assigned to β-sheet structures. For the spectra of the SF/HA hydrogels (Figure 5A), as the HA content increased, the broad peak including the alcohol group around 1040 cm^−1^ increased significantly. Since the silk fibroin protein has no strong vibrational mode within this region, this region can be established as an indicator of the peaks of the HA components in the SF/HA mixtures. Amide I and II regions (1700–1450 cm^−1^) of the SF and HA components overlap strongly because both silk and HA have vibrational modes in this region.

After loading curcumin into the SF and SF/HA hydrogels, none of the individual peaks belonging to curcumin were observed in the spectra obtained (Figure 5B–D). This is because the curcumin, SF and HA components strongly overlap having vibrational modes in this region [34]. Only for the highest proportions of curcumin (B5, C1 and C3), a peak is glimpsed at 1140 cm^−1^ that can be attributed to C-O-C stretching [44,45].

### 3.4. Cross-Sectional Morphology of the Hydrogels

**Figure 6:**
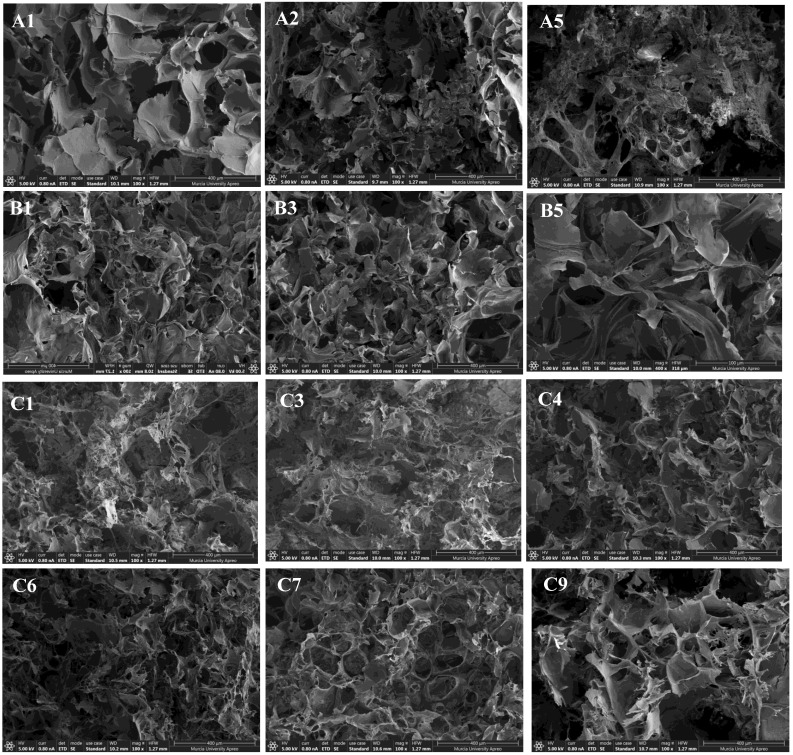
Observationof the morphology by FESEM of SF (A1,B1), SF/HA (A2,A5) and SF/curcumin hydrogels (B1,B3,B5); SF/HA/curcumin hydrogels with SF/HA = 80/20 (C1,C4,C7) and SF/curcumin = 40/60 (C3,C6,C9).

The microstructure, which can be observed by the FESEM technique, has a great impact on the properties of hydrogel, such as swelling behaviour, permeability, etc. [16]. The FESEM images shown in Figure 6 revealed that all cross-sectional samples had typically porous scaffold structures with interconnected pores. As can be seen, the appearance is quite similar, but some differences can be observed. Firstly, sample A1 (only SF) seems to show the largest pore size. This could be due to the incorporation of HA into the hydrogel matrix, which promotes the formation of porous structures, probably due to its high water-binding capacity [46]. A Similar conclusion was reached by Xiao et al. [16] and Yan et al. [1]. However, sample B1, which also has only SF, shows a similar pore size compared to the rest of samples containing HA. The differences found between samples A1 and B1 could be due to the fact that sample B1 was synthesised using NaOH, which promotes the formation of more and smaller pores in the SF hydrogel [47]. Secondly, the higher concentration of HA (without the presence of curcumin), i.e., A5 sample with 60 wt.% of HA, seems to lead to a more heterogeneous structure than sample A1 (0 wt.% of HA). In fact, the appearance of sample A5 may induce some kind of phase separation. However, compared to the samples with curcumin and similar content of HA, i.e., samples C6 and C9, we observed that a low concentration of curcumin (9 and 5 wt.%, respectively) gives surprisingly more homogeneous structures. This fact could suggest a synergistic effect between the three components (SF, HA, and curcumin).

### 3.5. Thermal Stability of the Hydrogels

**Figure 7:**
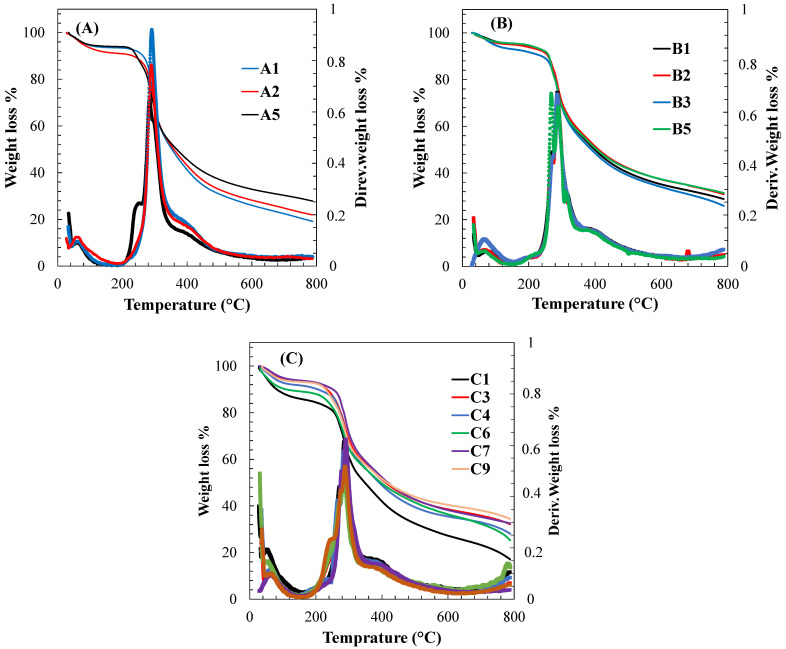
Thermogravimetric analysis (TGA) and differential thermal analysis (DTA): (**A**) SF/HA; (**B**) curcumin-loaded SF; and (**C**) curcumin-loaded SF/HA hydrogels.

Figure 7 displays the weight loss of different samples from room temperature to 800 °C, which reveals the degradation rates and average degradation temperatures of each component in the SF/HA, curcumin-loaded SF, and curcumin-loaded SF/HA hydrogels. As can be seen, all samples show at least three ranges. The first loss corresponds to the removal of bound water by evaporation of all samples at the temperature of 120 °C [48]. The water loss for pure SF ranged between 4.4 to 6.5 wt.% for B1 and A1, respectively. With the addition of HA, the percentage of bound water increases to 8.9% for sample A2 and 6.5% for A1. Sample C1 (curcumin-loaded SF/HA, 29/57/14) showed the highest amount of bound water (about 14 wt.%) [48]. Between 120 and 250 °C, all samples showed a stable weight loss. Above 250 °C, a change of a slope can be clearly observed, probably associated to the degradation of SF [49,50], this result is also confirmed in the DTA curves.

In addition, the DTA curves showed a main peak between 284 and 311 °C for sample B5 (curcumin-loaded SF) that can be attributed to thermal degradation of SF [51,52,53,54,55]. Two peaks at 247 °C and 253 °C occurred in the thermogram of samples A5 and C9, which may be related to the decomposition of unbound HA remaining in the composite hydrogel [49,50]. In addition, peaks at temperatures ranging from 266 °C to 277 °C could be considered thermal decomposition of curcumin [56,57,58]. Finally, above 350 °C the weight loss of approximately 17 to 32% corresponds to thermal decomposition of the residues.

### 3.6. X-ray Powder Diffraction

**Figure 8:**
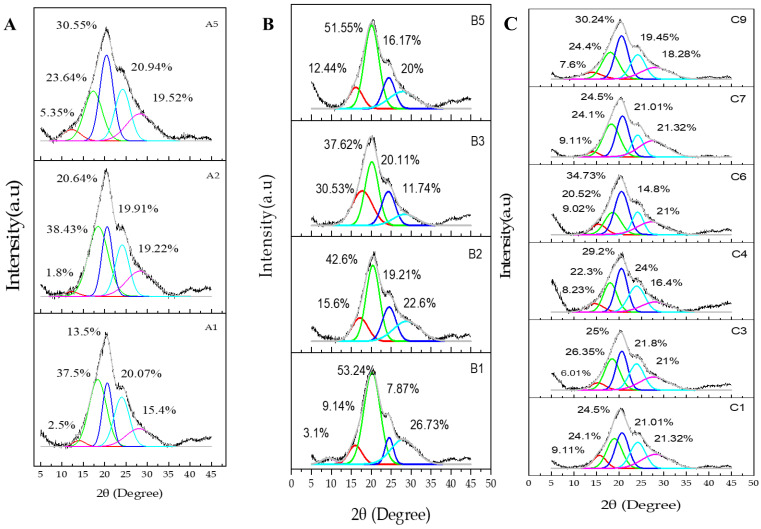
X-ray patterns of (**A**) SF/HA hydrogels; (**B**) curcumin-loaded SF; and (**C**) curcumin-loaded SF/HA hydrogels.

Figure 8 shows the X-ray diffraction of the SF, SF/HA, curcumin-loaded SF and curcumin-loaded SF/HA hydrogels. As can be seen in Figure 8A, A1 shows three crystal peaks when deconvoluted at 18.4, 20.64, and 24.1°, and a broad peak at 28.1°, and a smaller one at 14.2 indicating the coexistence of silk I and silk II [55,59], which also confirmed the two other samples (A2 and A5) with peaks at 12.2, 20.6, 24° and 12.3, 20.4, 24.22, 28.3°, respectively. Compared to SF hydrogel (see Supplementary Materials Table S1), the d-spacing and the area of the crystalline regions increased with slight shifts, denoting that the crystallinity of the SF/HA hydrogel decreased as the HA content increased, attributed to the interactions and good compatibility between SF and HA [60]. This result revealed that the complexation between SF and HA through high ultrasonic energy reduced the crystallinity [59].

Figure 8B represents the diffractogram of the SF hydrogel and the curcumin-loaded SF hydrogels. After deconvolution, it showed a strong peak at 20.2° and three smaller peaks at 9.2°, 16°, 24.5° for β-sheet, and another broad peak at 28.03° also assigned the existence of α-helix. On the other hand, the X-ray diffractogram of curcumin showed intense and narrow crystallinity peaks denoting a high crystallinity nature [56,61,62,63]. In the subsequent development, B2, B3, and B5 also describe the formation of silk I and silk II. Compared to B1, the peak at 9.2° disappeared, and the d-spacing and area of silk II increased as the curcumin content increased (see Supplementary Materials Table S2). These findings suggested that, in the presence of curcumin, SF may develop a structural transition from silk I to silk II [64]; with the existence of silk I. The disappearance of the crystalline peaks of curcumin [62,65], probably revealed the effective loading in the SF/CUR composite hydrogel [61,63], promoting the good solubility and bioavailability of curcumin [61,66].

The addition of curcumin to the SF/HA hydrogels also showed the formation of silk I and silk II (Figure 8) while the state of curcumin transitioned from crystalline to amorphous, which was determined by the disappearance of the crystalline peaks, and this is also confirmed by the d-spacing values (see Table S3 in the Supplementary Materials).

### 3.7. Swelling Properties

The Table 2 presents the swelling ration and the water uptake of the differents samples. As can be showed that all the hydrogels obtained presented a water uptake higher than 85%.

Regarding the swelling results, samples C3 and C6, with a SF/HA ratio of 40/60 have the best water content and swelling properties (Table 2). These results may be related to the size and homogeneity of the hydrogel pores.

### 3.8. Study of the Kinetic Release of Curcumin from Different Hydrogels

**Figure 9:**
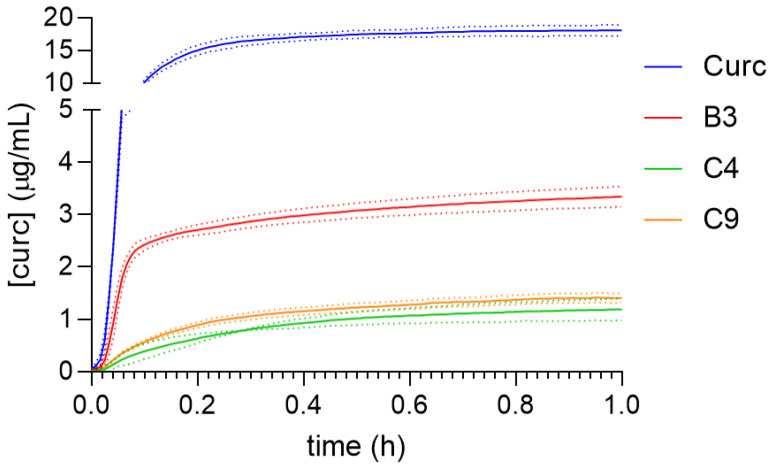
In vitro drug release profiles of curcumin powder and curcumin-loaded hydrogel made of SF and SF/HA with different mass ratio.

All release studies were performed under sink conditions after determining the solubility of curcumin (Curc) in the respective release medium. Figure 9 shows the release profiles of free curcumin and the different curcumin-loaded hydrogels. It can be seen that in all cases, the release profile follows first-order release kinetics [9,25] with a burst release phase and a plateau phase. Free curcumin powder reaches the plateau at 0.2 h, while sample B3, composed only of SF reaches the plateau at 0.1 h. The hydrogels containing HA, C4 and C9, reach the plateau later, 0.5 h. In all cases, the hydrogels despite their composition, delay the releases of curcumin with respect to the free powder. The addition of HA in the compositions studied (20 and 60%) hindered the release of curcumin compared to the pure SF hydrogels sample. Pure HA hydrogels have been reported to exhibit slower release of vanillic acid and Epimedin C than the SF/HA compositions [24]. While a small percentage of HA (5%) accelerated the release of vascular endothelial growth factors [67]. Samples C4 and C9 did not show a significant difference despite their SF:HA composition.

It was reported [44] that SF without antibacterial agent does not exhibit antibacterial activity, however, SF composite films containing curcumin exhibited high inhibition ratios during 12 h of incubation against *Staphylococcus aureus* (*S. aureus*; *G+*) bacteria, which are common bacteria found in wound infections [44]. Given the sustained release profile of SF/curcumin and SF/HA/curcumin composite hydrogels, a diffusion release is expected to be maintained for hours to constitute an effective bacterial barrier.

### 3.9. Cell Viability in Hydrogels

**Figure 10:**
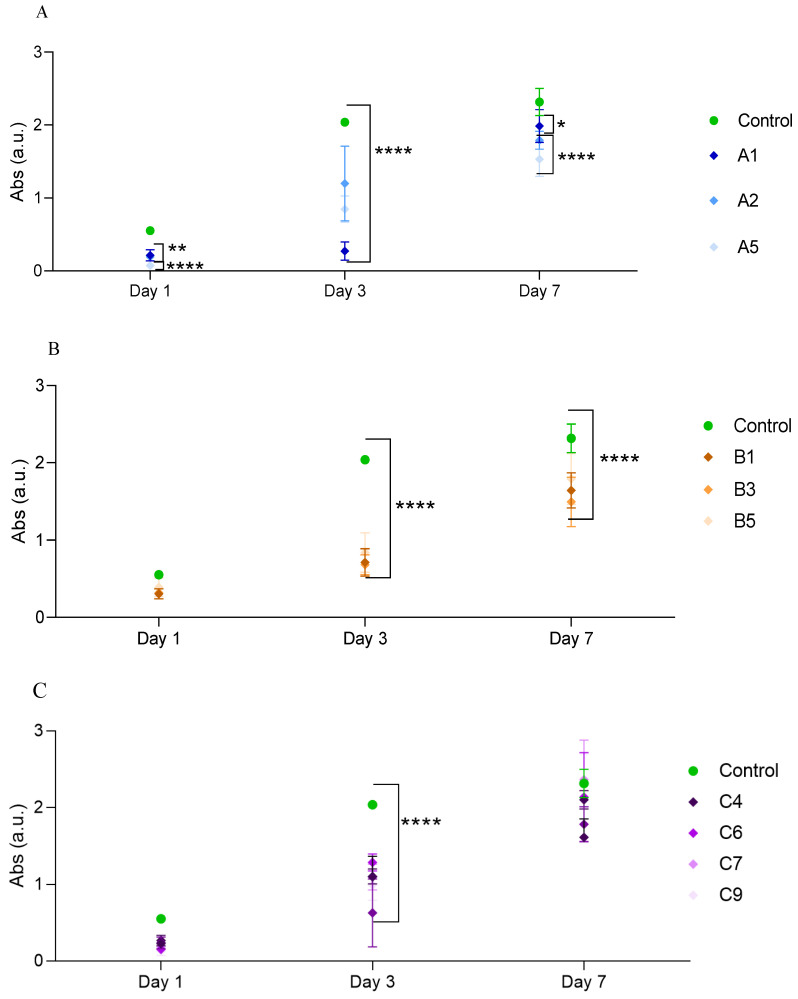
Cell density of (**A**) SF/HA; (**B**) curcumin-loaded SF; (**C**) curcumin-loaded SF/HA. Data are expressed as percentage of cell viability ± SD vs. concentration. * Indicates *p* < 0.05, ** indicates *p* < 0.01, and **** indicates *p* < 0.0001, compared to control.

**Figure 11:**
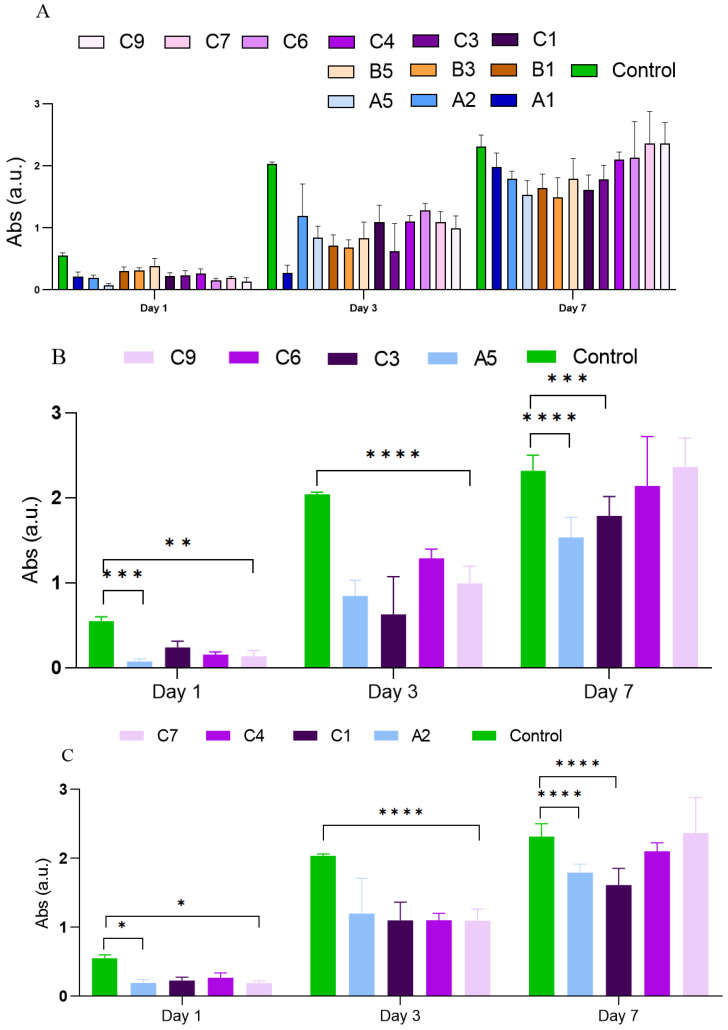
Comparative results of cell density between (**A**) SF/HA (A1, A2 and A5), curcumin-loaded SF (B1, B3 and B5), and curcumin-loaded SF/HA hydrogels (C1, C3, C4, C6 and C7; (**B**) SF/HA (A5) and curcumin-loaded SF/HA hydrogels (C3 C6, and C9)); (**C**) SF/HA (A2) and curcumin-loaded SF/HA hydrogels (C1 C4, and C7). Data are expressed as percentage of cell viability ± SD vs. concentration. * Indicates *p* < 0.05, ** indicates *p* < 0.01, *** indicates *p* < 0.001 and **** indicates *p* < 0.0001, compared to control.

The MTT absorbance of the L929 cell line seeded into the hydrogels was used to assess cell viability in the hydrogels. The main objective of this research is to discover the importance of curcumin in the SF/HA hydrogel for L929 cell development, for 1, 3 and 7 days. Figure 10 and Figure 11 represent the evaluation of cell viability of curcumin-loaded SF/HA versus SF/HA and curcumin-loaded SF hydrogels.

In Figure 10A, all samples showed good initial cell viability after one day, several L929 cells developed in the samples and over seven days, cells continued to survive and grow in the samples. Differences in proliferative activity between the control and A1 were significant (* *p* < 0.05), as well as between control and A2 and A5 (**** *p* < 0.0001). In addition, the low HA content A2 hydrogel tended to have more cells than the high HA content A5 hydrogel. The results are in agreement with those obtained by Xiao Hu et al. [48] that after 10 days of incubation of hMSCs in various silk/HA hydrogels, the trend of cell viability varied, with cell viability decreasing with increasing HA concentration.

Figure 10B shows the cytotoxicity of the curcumin SF-loaded scaffold on L929 cells. The control had a high cell proliferation rate point (**** *p* < 0.0001) compared to SF and curcumin-loaded SF, but the curcumin SF-loaded scaffold showed no toxicity during the 7 days of incubation and cell viability increased with increasing curcumin concentration. This result confirms previous findings from several works [25,68,69] that curcumin improved cell viability. The crucial point is that cell proliferation does not stop abruptly or slow down dramatically during the period.

The curcumin-loaded SF/HA hydrogel shows high cell viability, rapid growth and good compatibility results on the seventh day compared to the first day, furthermore, cell viability goes from a highly significant point (**** *p* < 0.0001) on day 3 to non-significant on day 7, where C6 and C9 are almost identical to the control (Figure 10C). These results suggest that adding curcumin to SF/HA positively modifies the tendency of cells within the SF/HA scaffold, which is also clear in Figure 10B,C.

As an epilogue, MTT values of the cells measured for the curcumin-loaded SF/HA scaffolds were remarkably higher than those for the SF/HA and curcumin-loaded SF, as shown in Figure 11A. The MTT viability experiment demonstrated how adding CUR to the SF/HA hydrogel to create curcumin-loaded SF/HA scaffolds boosted the proliferation of the L929 cell line compared to SF/HA hydrogels (Figure 11B,C), highlighting that the concentration of CUR is an important factor to consider. This result is mainly attributed to the good effect of curcumin on cell viability in the SF/HA scaffold.

### 3.10. Cell Morphology

**Figure 12:**
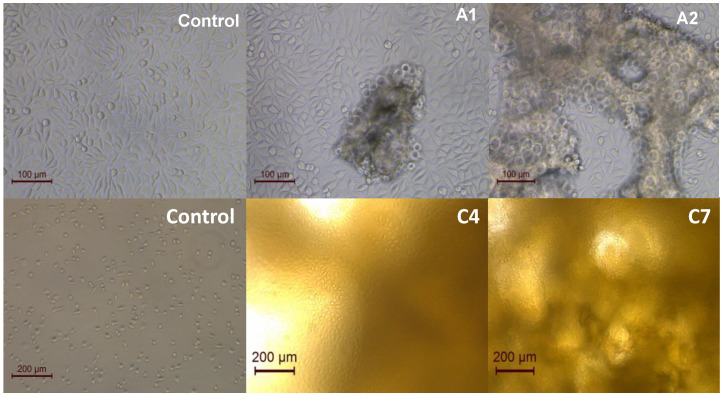
Morphological images of cells in the samples of Control, SF/HA (A1,A2) at three days and curcumin-loaded SF/HA hydrogels (C4,C7) at seven days.

L929 cells were observed microscopically to check the growth status of cells cultured with the hydrogels. Figure 12 shows that cells cultured with the SF and SF/HA hydrogels (A1 and A2) experienced a slight decrease in growth at 3 days with respect to the control group, while those cultured with the curcumin-loaded SF/HA hydrogels (C4 and C7), observed at 7 days, showed similar morphology and growth in the hydrogel as the control group, corroborating the cell viability results and perspective of these hydrogels as a wound healing therapy.

L929 cells were observed microscopically to check the growth status of cells cultured with the hydrogels. Figure 12 displays that cells cultured with the SF and SF/HA hydrogels (A1 and A2) experienced a slight decrease in growth at 3 days with respect to the control group, while those cultured with the curcumin-loaded SF/HA hydrogels (C4 and C7), observed at 7 days, showed similar morphology and growth in the hydrogel as the control group, corroborating the cell viability results and perspective of these hydrogels as a wound healing therapy.

## 4. Conclusions

In this study, a simple, fast, and non-toxic method, sonication, was used to prepare hydrogel for biomedical application. SF/HA hydrogel, curcumin-loaded SF and curcumin-loaded SF/HA were prepared and characterised by Fourier transform infrared spectroscopy (FTIR), X-ray diffraction (XRD), thermogravimetric analysis (TGA) and field emission scanning electron microscopy (FESEM). This was done to ensure that the curcumin in the SF/HA hydrogel was adequately loaded to improve its therapeutic properties. The main objective of this study was achieved, as can be seen from the cell viability test which clearly showed that curcumin improves the therapeutic characteristic of SF/HA. These results suggest that the recently prepared scaffold, with its low cytotoxicity, excellent biocompatibility. In addition, curcumin-loaded nanofibrous scaffolds have been evaluated for drug release, antioxidant, antimicrobial and anti-inflammatory activities in vitro. The results showed that curcumin exhibited sustained release behavior from the nanofibrous scaffolds and maintained its free radical scavenging ability, and these scaffolds effectively inhibited the growth of *S. aureus* (>95%). Therefore, it is expected in vivo assays that curcumin-loaded SF/HA hydrogels exhibit a synergistic effect as to be excellent potential candidates for wound dressings and tissue engineering scaffolds. Furthermore, previous studies [43] evaluated the in vitro antioxidant, antimicrobial and anti-inflammatory activities of curcumin-loaded P(LLA_CL) fibroin nanofibrous scaffolds. The results showed that curcumin exhibited sustained release behavior from the nanofibrous scaffolds and maintained its free radical scavenging ability, and these scaffolds effectively inhibited the growth of *S. aureus* (> 95%). Cell viability studies have shown that the addition of CUR to the SF/HA hydrogel enhances the proliferation of the L929 cell line compared to hydrogels that do not contain CUR, highlighting that the presence and concentration of CUR is an important factor for biomedical applications. Therefore, it is expected that in in vivo assays curcumin-loaded SF/HA hydrogels obtained in this work will show a synergistic effect as to be excellent potential candidates for wound dressings and tissue engineering scaffolds. In vivo assays will constitute the next step of this work.

## Figures and Tables

**Table 1:** Composition and nomenclature of the curcumin-loaded SF/HA hydrogels prepared.

Sample Code	SF/HA (wt./wt.%)	SF/Curcumin (wt./wt.%)	SF (wt.%)	HA (wt.%)	Curcumin (wt.%)
A1	100/0	0/0	100	0	0
A2	80/20	0/0	80	20	0
A3	60/40	0/0	60	40	0
A4	50/50	0/0	50	50	0
A5	40/60	0/0	40	60	0
B1	100/0	0/0	100	0	0
B2	100/0	16/1	94	0	6
B3	100/0	8/1	89	0	11
B4	100/0	4/1	80	0	20
B5	100/0	2/1	67	0	33
C1	80/20	2/1	57	14	29
C2	50/50	2/1	40	40	20
C3	40/60	2/1	33	50	17
C4	80/20	4/1	67	17	17
C5	50/50	4/1	44	44	11
C6	40/60	4/1	36	55	9
C7	80/20	8/1	73	18	9
C8	50/50	8/1	47	47	6
C9	40/60	8/1	38	57	5

A1 and B1 samples have the same composition but have been prepared following different protocols.

**Table 2:** Swelling properties of the different hydrogels sample obtained.

Sample Code	Swelling Ratio (g/g)	Water Uptake (%)
B1	6.10	85.91
B2	7.79	88.62
B3	7.83	88.67
B5	6.21	86.13
C1	4.539	81.94
C3	9.46	90.44
C6	11.48	91.99

## Supplementary Materials

The following supporting information can be downloaded at: https://www.mdpi.com/article/10.3390/polym15030504/s1, Table S1: X-ray diffraction of SF/HA hydrogels; Table S2: X-ray diffraction of curcumin-loaded SF; Table S3: X-ray diffraction of curcumin-loaded SF/HA hydrogels.
