# Supplementary material for: Accelerated Simple Preparation of Curcumin-Loaded Silk Fibroin/Hyaluronic Acid Hydrogels for Biomedical Applications
Source: Polymers (Basel). 2023 Jan 18;15(3):504. doi: 10.3390/polym15030504 (PMC9919302; doi:10.3390/polym15030504)
Supplement: Supplementary file 1 [file polymers-15-00504-s001.zip › polymers-2119931-supplementary.pdf]

Supporting information

Accelerated simple preparation of curcumin-loaded silk fibroin/hyaluronic acid hydrogels for wound healing applications

Mohamed Chaala [1], Fatima Zohra Sebba [1], Marta G. Fuster [2], Imane Moulefera [2,*], Mercedes G. Montalbán [2], Guzmán Carissimi [2] and Gloria Víllora [2]

[1] Laboratoire de Chimie Physique Macromoléculaire, Département de Chimie, Université Oran1 Ahmed Ben Bella, B.P 1524, El-Menaouer 31000, Oran, Algeria ; chaalamohamed07@gmail.com ; fzsebba@yahoo.fr.

[2] Chemical Engineering Department, Faculty of Chemistry, Regional Campus of International Excellence "Campus Mare Nostrum", University of Murcia, 30071 Murcia, Spain; marta.g.f@um.es; mercedes.garcia@um.es; guzmancarissimi@gmail.com; gvillora@um.es

* Correspondence: Tel: +34 868 88 7394 imane.moulefera@um.es

Table S1. X-ray diffraction of SF/HA hydrogels.

**A1**	2Theta	14.02	18.40	20.64	24.01	28.10
	d-Spacing d(hkl) Å	6.31	4.82	4.29	3.70	3.17
	Peak area	0.19	2.92	1.57	1.91	1.19
**A2**	2Theta	12.20	18.50	20.60	24.12	28.35
	d-Spacing d(hkl) Å	7.249	4.79	4.31	3.69	3.14
	Peak area	0.15	3.12	1.68	1.62	1.56
**A5**	2Theta	12.32	17.34	20.45	24.22	28.30
	d-Spacing d(hkl) Å	7.179	5.11	4.34	3.67	3.15
	Peak area	0.51	2.23	2.89	1.98	1.84

Table S2. X-ray diffraction of curcumin-loaded SF.

**B1**	2Theta	9.20	16	20.20	24.50	28.03
	d-Spacing d(hkl) Å	9.60	5.53	4.39	3.63	3.18
	Peak area	0.27	0.83	4.86	0.71	2.43
**B2**	2Theta		17.16	20.40	24.50	28.96
	d-Spacing d(hkl) Å		5.164	4.35	3.63	3.08
	Peak area		1.28	3.51	1.58	1.86
**B3**	2Theta		17.75	20.14	24.30	28.48
	d-Spacing d(hkl) Å		4.99	4.40	3.66	3.13
	Peak area		2.38	2.94	1.57	0.91

**B5**	2Theta		16.13	20.10	24.40	27.92
	d-Spacing d(hkl) Å		5.49	4.41	3.64	3.19
	Peak area		0.98	4.09	1.28	1.57

Table S3. X-ray diffraction of curcumin-loaded SF/HA hydrogels.

**C1**	2Theta	15.70	19	20.70	24.20	28.25
	d-Spacing d(hkl) Å	5.64	4.67	4.29	3.67	3.16
	Peak area	0.78	2.06	2.09	1.79	1.82
**C3**	2Theta	15.3	18.50	20.65	23.92	27.80
	d-Spacing d(hkl) Å	5.79	4.79	4.29	3.72	3.21
	Peak area	0.48	2.12	2.01	1.75	1.68
**C4**	2Theta	14.6	18.05	20.57	23.95	28.20
	d-Spacing d(hkl) Å	6.06	4.91	4.31	3.71	3.16
	Peak area	0.66	1.78	2.33	1.92	1.31
**C6**	2Theta	15.46	18.61	20.59	24.18	27.28
	d-Spacing d(hkl) Å	5.72	4.763	4.308	3.678	3.27
	Peak area	0.708	1.61	2.73	1.16	1.65
**C7**	2Theta	14.30	18.32	20.79	24.18	27.58
	d-Spacing d(hkl) Å	6.187	4.84	4.27	3.68	3.23
	Peak area	0.778	2.06	2.09	1.79	1.82
**C9**	2Theta	13.83	18.09	20.60	24.20	28.14
	d-Spacing d(hkl) Å	6.39	4.89	4.30	3.67	3.16
	Peak area	0.63	2.02	2.50	1.61	1.51
